# Cardiovascular magnetic resonance of mitral valve length in hypertrophic cardiomyopathy

**DOI:** 10.1186/s12968-016-0250-5

**Published:** 2016-06-04

**Authors:** Mika Tarkiainen, Petri Sipola, Mikko Jalanko, Tiina Heliö, Mika Laine, Vesa Järvinen, Kaisu Häyrinen, Kirsi Lauerma, Johanna Kuusisto

**Affiliations:** Department of Radiology, Kuopio University Hospital, Kuopio, Finland; Heart and Lung Center, Department of Cardiology, Helsinki University Central Hospital, Helsinki, Finland; HUS Medical Imaging Center, Clinical Physiology and Nuclear Medicine, Hyvinkää Hospital, Hyvinkää, Finland; University of Eastern Finland, Kuopio, Finland; Centre for Medicine and Clinical Research, University of Eastern Finland and Kuopio University Hospital, Kuopio, Finland; Department of Radiology, Helsinki University Central Hospital, Helsinki, Finland

**Keywords:** Cardiomyopathy, Hypertrophic, Mitral valve, Cardiovascular magnetic resonance

## Abstract

**Background:**

Previous data suggest that mitral valve leaflets are elongated in hypertrophic cardiomyopathy (HCM), and mitral valve leaflet elongation may constitute a primary phenotypic expression of HCM. Our objective was to measure the length of mitral valve leaflets by cardiovascular magnetic resonance (CMR) in subjects with HCM caused by a Finnish founder mutation in the myosin-binding protein C gene (MYBPC3-Q1061X), carriers of the same mutation without left ventricular hypertrophy, as well as in unselected consecutive patients with HCM, and respective controls.

**Methods:**

Anterior mitral valve leaflet (AML) and posterior mitral valve leaflet (PML) lengths were measured by CMR in 47 subjects with the Q1061X mutation in the gene encoding MYBPC3 and in 20 healthy relatives without the mutation. In addition, mitral valve leaflet lengths were measured by CMR in 80 consecutive non-genotyped patients with HCM in CMR and 71 age- and gender-matched healthy subjects.

**Results:**

Of the subjects with the MYBPC-Q1016X mutation, 32 had left ventricular hypertrophy (LVH, LV maximal wall thickness ≥ 13 mm in CMR) and 15 had no hypertrophy. PML was longer in patients with the MYBPC3-Q1061X mutation and LVH than in controls of the MYBPC group (12.8 ± 2.8 vs 10.6 ± 1.9 mm, *P* = 0.013), but the difference between the groups was not statistically significant when PML was indexed for BSA (*P* = 0.066), or when PML length was adjusted for BSA, age, gender, LV mass and ejection fraction (*P* = 0.195). There was no significant difference in the PML length in mutation carriers without LVH and controls (11.1 ± 3.4 vs 10.6 ± 1.9, *P* = 0.52). We found no difference in AML lengths between the MYBPC mutation carriers with or without hypertrophy and controls. In 80 consecutive non-genotyped patients with HCM, there was no difference either in AML or PML lengths in subjects with HCM compared to respective control subjects.

**Conclusions:**

In subjects with HCM caused by the Q1061X mutation in the MYBPC3 gene, the posterior mitral valve leaflets may be elongated, but mitral valve elongation does not constitute primary phenotypic expression of the disease. Instead, elongated mitral valve leaflets seem to be associated with body size and left ventricular remodeling.

## Background

Hypertrophic cardiomyopathy (HCM) is the most common genetic cardiomyopathy with an estimated prevalence of 1:500 in the general population [1]. It is also the most common cause of sudden cardiac death among young people and athletes [2]. HCM is inherited as an autosomal dominant trait and at the moment over 1400 mutations in at least 13 different HCM-causing genes encoding mainly cardiac sarcomeric proteins have been identified [2]. The penetrance of mutations is highly variable and incomplete [3]. Left ventricular hypertrophy (LVH) in HCM usually develops during adolescence, but particularly myosin binding protein C (MYBPC3) mutations are slower to manifest and mutation carriers often demonstrate onset of hypertrophy later in life [4].

Mutations in the cardiac myosin-binding protein C (*MYBPC3*) gene are a common cause of HCM. It is estimated that 42 % of the mutations causing HCM are found in the *MYBPC3* gene [5]. The most common single mutation responsible for HCM in Finland is the founder mutation Q1061X in the *MYBPC3* gene (*MYBPC3*-Q1061X). It accounts for approximately 11 % [6] of the Finnish HCM cases [6, 7]. This mutation is characterized by a relatively low penetrance, and it is rare outside Finland. In patients with *MYBPC3*-Q1061X, marked hypertrophy and significant arrhythmias may occur, but in most cases the phenotype is mild [6, 8].

Several traditional echocardiographic studies have reported that mitral valve is elongated in HCM, particularly in the obstructive form of the disease [9–12]. Quantitative characterization of mitral valve length by echocardiography is, however, inaccurate. Cardiovascular magnetic resonance (CMR) may provide a more accurate method to characterize mitral valve, but so far, there are no generally accepted normal values for mitral valve leaflet lengths measured by CMR. In the study by Maron et al., patients with HCM had significantly longer anterior and posterior mitral valve leaflets (AML and PMLs, respectively) compared to controls. Moreover, elongated anterior mitral valve leaflets seemed to represent a primary phenotypic expression of HCM, as subjects with HCM-causing sarcomeric mutations but no LVH had elongated AMLs [13]. Recently, also Captur et al. reported two CMR studies suggesting that elongated AML is a feature of sarcomeric mutation induced subclinical HCM [14, 15], and Reant et al. found longer AMLs in G+/LVH- subjects compared to controls in their study [16].

Therefore, as the knowledge of mitral valve length in clinical and subclinical HCM is quite limited, the aim of the present study was to investigate mitral valve leaflet length in carriers of the single *MYBPC3*-Q1016X mutation, with and without LVH and mutation negative relatives without hypertrophy by CMR. In addition, we measured mitral valve leaflets in CMR images of 80 consecutive non-genotyped patients with HCM criteria in CMR, and matched controls with normal CMR.

## Methods

### Study population and echocardiography

In the MYBPC study, we screened 74 adult individuals from 25 families carrying the Finnish founder mutation *MYBPC3*-Q1061X for inclusion at the University Hospitals of Helsinki and Kuopio. We prospectively invited all available subjects with *MYBPC3*-Q1061X mutation (G+) and their healthy relatives without mutation (G-) from Kuopio and Helsinki University Hospital areas, and included all who were willing to participate in the study. The genetic diagnosis was performed at the Genome Center of the University of Eastern Finland as previously described [6]. Seven screened individuals were excluded from the study due to having a pacemaker or implantable cardioverter defibrillator (ICD). The final study population included 47 subjects with the *MYBPC3*-Q1061X mutation, and 20 healthy relatives without the *MYBPC3*-Q1061X mutation from the same families. Echocardiographic studies were performed by experienced cardiologists with Vivid 7 (GE Vingmed, Norway) ultrasound equipment and analyzed with Echopac software (GE Vingmed, version 10.0.1, Norway). Mitral regurgitation was determined and graded by color Doppler. Left ventricular outflow tract (LVOT) maximal flow velocity was measured by continuous wave Doppler (gradient > 30 mmHg on echocardiography considered significant).

The G+/LVH+ group of the MYBPC study consisted of 32 patients with *MYBPC*-Q1061X mutation and significant hypertrophy consistent with HCM phenotype (LV maximal wall thickness ≥ 13 mm in CMR). The G+/LVH- group consisted of 15 subjects with *MYBPC3*-Q1061X mutation and no HCM phenotype (LV maximal wall thickness < 13 mm in CMR). The control population consisted of 20 healthy relatives without the *MYBPC3*-Q1061X mutation. The local ethics committees of the University Hospitals of Helsinki and Kuopio approved the study protocol and all subjects gave prior written consent.

In the Archive study, of all 80 consecutive unselected individuals with HCM criteria (LV maximal wall thickness ≥ 13 mm, in the absence of other causes for LVH, such as aortic stenosis or hypertensive heart disease) in CMR diagnosed by referred radiologist at the Kuopio University Hospital from April 2005 to June 2012, were included in the Archive HCM group. In addition, 71 individuals with normal CMR findings, who were matched with patients with HCM in the Archive substudy with respect to age and sex in unpaired fashion, were obtained from the Kuopio University Hospital picture archiving and communication system (PACS) to be included in the Archive control group. In Archive-HCM patients, genetic cause of the disease was not systematically defined, although some of the patients may have been detected to have one of the three Finnish founder mutations causing HCM [6–8]. In archive patients, CMR was performed as a part of clinical patient care and consequently, no consent was obtained. The ethics committee of the Kuopio University Hospital accepted the use of CMR data of archive patients as a part of the present study (17/4/2012).

### CMR

In the MYBPC study, CMR was performed prospectively according to the same study protocol in two participating university hospitals (Kuopio University Hospital and Helsinki University Hospital). Imaging was performed by using a 1.5 T scanner (Magnetom Avanto; Siemens Medical Solutions, Erlangen, Germany) and a body-array coil in both participating hospitals. After scout images were obtained, 8-mm sections with retrospectively ECG-gated steady-state, free precession breath-hold cine images in 3 standard long-axis planes (4-, 3- and 2-chamber views) and sequential 8-mm short axis slices from atrium to apex with an intersection gap of 20 % were acquired. The typical parameters used to perform cine CMR were as follows: 48/1.1 (repetition time msec/echo time msec), a 65° flip angle, a 192 × 256 matrix, and a 280–360-mm field of view.

In the Archive group, all consecutive unselected subjects were imaged as part of the clinical work with the same scanner and with the same imaging protocol, planes and sequences as in the MYBPC study.

### Image analyses

**Fig. 1:**
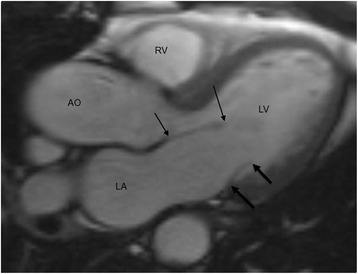
Measurement of mitral valve leaflets in 3-chamber diastolic image. Anterior mitral leaflet (AML) is indicated in thin arrows and posterior mitral leaflet (PML) in thick arrows. RV = right ventricle, LV = left ventricle, AO = aorta, LA = left atrium

Image analysis was performed by using Sectra IDS7/dx workstation. The radiologists analyzing CMR images were blinded to genetic and clinical findings of the study subjects. The lengths of anterior mitral leaflet (AML) and posterior mitral leaflet (PML) were measured in the cine 3-chamber view image, from the most distal part of the leaflet to its insertion in mid- or end-diastole, using the last diastolic image where the MV was clearly visible (Fig. 1) [13, 17, 18]. One radiologist (M.T., with 5 year experience in CMR) performed all mitral valve length measurements on MR images. LVMWT was measured at end-diastole in the short-axis orientation in all 67 subjects of the MYBPC study by one radiologist (K.L.) with over 20 year experience in CMR, and by clinical radiologists in the Archive study. To evaluate left ventricular ejection fraction (LVEF), left ventricular end-diastolic volume (LVEDV), left ventricular mass (LVM) and left ventricular end-systolic volume (LVESV), the endocardium and the epicardium were manually traced, with the papillary muscles and trabeculations excluded. LVESV was analyzed from the cine images with smallest LV cavity. The other measurements were performed of the first image after R-wave. LV analyses were performed with dedicated software (Argus, Siemens, Erlangen, Germany) [19] by a single radiologist (M.T.) in the MYBPC study, and by clinical radiologists in the Archive study. Body surface area (BSA) was calculated by using Du Bois method.

To evaluate mitral valve leaflet length measurement repeatability, mitral valve leaflets were re-measured in 20 randomly selected subjects with the MYBPC3-Q1061X mutation. To study intraobserver variability, one radiologist (M.T.) measured mitral valve leaflet lengths twice, with at least 3 months between the measurements. To test interobserver variability, second observer (K.H.) repeated the same measurements in the same set of 20 subjects.

### Statistical analyses

Baseline continuous data are expressed as mean ± SD. As many of the variables studied were not normally distributed, they were compared with Kruskal-Wallis one-way analysis of variance (the MYBPC-group) and independent samples Mann-Whitney *U* test (the Archive-group), when appropriate. Adjustments were done by using general linear model. Spearman correlation coefficients were used to study the association of AML and PML lengths with clinical, echocardiographic and CMR findings. Paired samples *T*-test was used for comparing age and sex matched G+/LVH- and controls and also in repeatability testing. Intraclass correlation coefficients (ICC) were determined. SPSS 19.0 (SPSS Inc, Chicago, IL, USA) was used for statistical analyses. A *P* value less than 0.05 was considered significant.

## Results

### Clinical, echocardiographic and CMR characteristics

Clinical, echocardiographic and CMR findings of the study subjects are presented in Table 1. In the MYBPC group, G+/LVH- subjects were younger than G+/LVH+ subjects and controls, as expected and had also lower BSA. There were more male subjects in the G+/LVH+ group than in the G+/LVH- and control groups. Body surface area (BSA) was lower in the G+/LVH- group compared to other groups.

**Table 1:** Clinical, echocardiographic and cardiac MRI (CMR) findings in the MYBPC group and in the Archive group

	MYBPC control	MYBPC G+/LVH-	MYBPC G+/LVH+	*P*	Archive control	Archive HCM	*P*
Patients, n	20	15	32		71	80	
Age, y	46 ± 17	33 ± 16	50 ± 11	.007*	53 ± 14	55 ± 16	.42
Men, n	5 (25 %)	3 (20 %)	20 (63 %)	<.001*	44 (62 %)	51 (64 %)	.82
BSA, m^2^	1.87 ± 0.28	1.75 ± 0.18	1.94 ± 0.20	.004*	1.88 ± 0.22	1.90 ± 0.21	.44
Height, cm	169 ± 10	167 ± 6	174 ± 9	.040*	171 ± 10	170 ± 8	.718
Weight, kg	76 ± 24	68 ± 13	81 ± 14	.005*	77 ± 16	80 ± 16	.248
BMI	27 ± 8	24 ± 4	27 ± 4	.129	26 ± 4	28 ± 5	.141
NYHA, n							
I	20 (100 %)	15 (100 %)	28 (88 %)		N.A.	N.A.	
II	-	-	4 (13 %)		N.A.	N.A.	
LVOT gradient, mmHg	7.1 ± 2.9	5.7 ± 1.8	8.5 ± 11.4	.42	N.A.	N.A.	
LVMWT, mm	10.2 ± 2.8	9.5 ± 1.6	22.1 ± 5.7	<.001*	10.5 ± 2.5	19.2 ± 4.5	<.001*
LVMI, g/m2	45 ± 9	49 ± 13	68 ± 21	<.001*	N.A.	N.A.	
LVEDVI, ml/m2	78 ± 13	78 ± 15	74 ± 14	.55	75 ± 17	74 ± 22	.49
LVESVI, ml/m2	32 ± 10	30 ± 7	28 ± 10	.13	33 ± 9	31 ± 15	.019*
LVEF, %	60 ± 8	62 ± 5	63 ± 9	.12	55 ± 7	59 ± 10	.007*
AML, mm (range)	25.0 ± 2.9 (20–30)	24.7 ± 3.8 (19–33)	24.8 ± 4.1 (19–42)	.81	25.1 ± 3.7 (16–36)	25.3 ± 3.9 (17–41)	.66
PML, mm (range)	10.6 ± 1.9 (8–15)	11.1 ± 3.4 (6–16)	12.7 ± 2.8 (9–22)	.056	14.4 ± 3.6 (7–24)	14.2 ± 3.5 (8–25)	.55
AML index, mm/m2	13.7 ± 2.2	14.2 ± 2.2	12.9 ± 2.5	.034*	13.5 ± 2.4	13.8 ± 2.5	.68
PML index, mm/m2	5.8 ± 1.2	6.2 ± 1.5	6.6 ± 1.5	.214	7.8 ± 2.2	7.6 ± 1.9	.90

*MYBPC-control* healthy controls without MYBPC mutation, *MYBPC G+/LVH-* MYBPC mutation carriers without LVH, *MYBPC G+/LVH+* MYBPC mutation carriers with LVH, *Archive control* control subjects with normal CMR findings, *Archive HCM* subjects with HCM in CMR, *BSA* body surface area, *BMI* body mass index, *NYHA* New York Heart Association functional class, *LVOT gradient* left ventricular outflow tract gradient, *LVMWT* left ventricular maximal wall thickness, *LVMI* left ventricular mass index, *LVEDVI* left ventricular end-diastolic volume index, *LVESVI* left ventricular end-systolic volume index, *LVEF* left ventricular ejection fraction, *AML* anterior mitral leaflet length, *PML* posterior mitral leaflet length, *AML index* AML indexed for BSA, *PML index* PML indexed for BSA, *N.A.* not available. * Significance *P* < 0.05

None of the study subjects of the MYBPC study had a history of surgical septal myectomy or alcohol septal ablation, or mitral valve prolapse or significant mitral regurgitation on echocardiography. Altogether, 18 study subjects had insignificant grade 1/4 mitral regurgitation. There was one patient with significant left ventricular outflow tract obstruction (gradient > 30 mmHg on echocardiography) in the MYBPC G+/LVH+ group, but there were no significant difference in the mean LVOT gradients between the three MYBPC study groups. In CMR, there were expected differences between the MYBPC subgroups in LVMWT and in left ventricular mass index (LVMI), but no significant differences in ventricular volume indices (LVEDVI and LVESVI) or in LVEF between the three MYBPC study groups. In CMR, LVH in the G+/LVH+ patients was mostly quite moderate, with asymmetric distribution in the anteroseptal wall. Of G+/LVH+ patients, 72 % had septal and 25 % anterior wall hypertrophy. None of the subjects had true apical hypertrophy limited to apex.

In the Archive group, there was no significant difference in age, gender distribution or BSA between patients with HCM and controls. There was expected difference in LVMWT between the patients with HCM and controls. In addition, LVESVI was smaller and LVEF higher in the HCM group compared to the respective control group.

### Mitral valve leaflet lengths

**Fig. 2:**
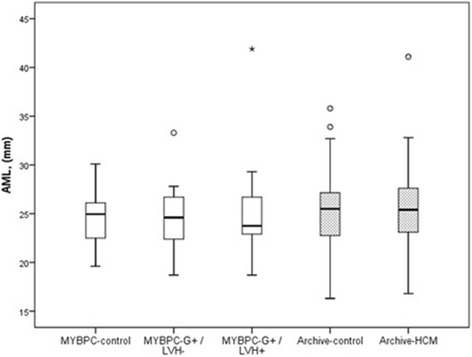
Anterior mitral valve leaflet (AML) lengths in study groups. The lower edge of the box presents 25th percentile and the upper edge 75th percentile. A line across the box is the median. The small circles present outliers and asterisk extreme value. There is no significant difference between the three and two groups, respectively

**Fig. 3:**
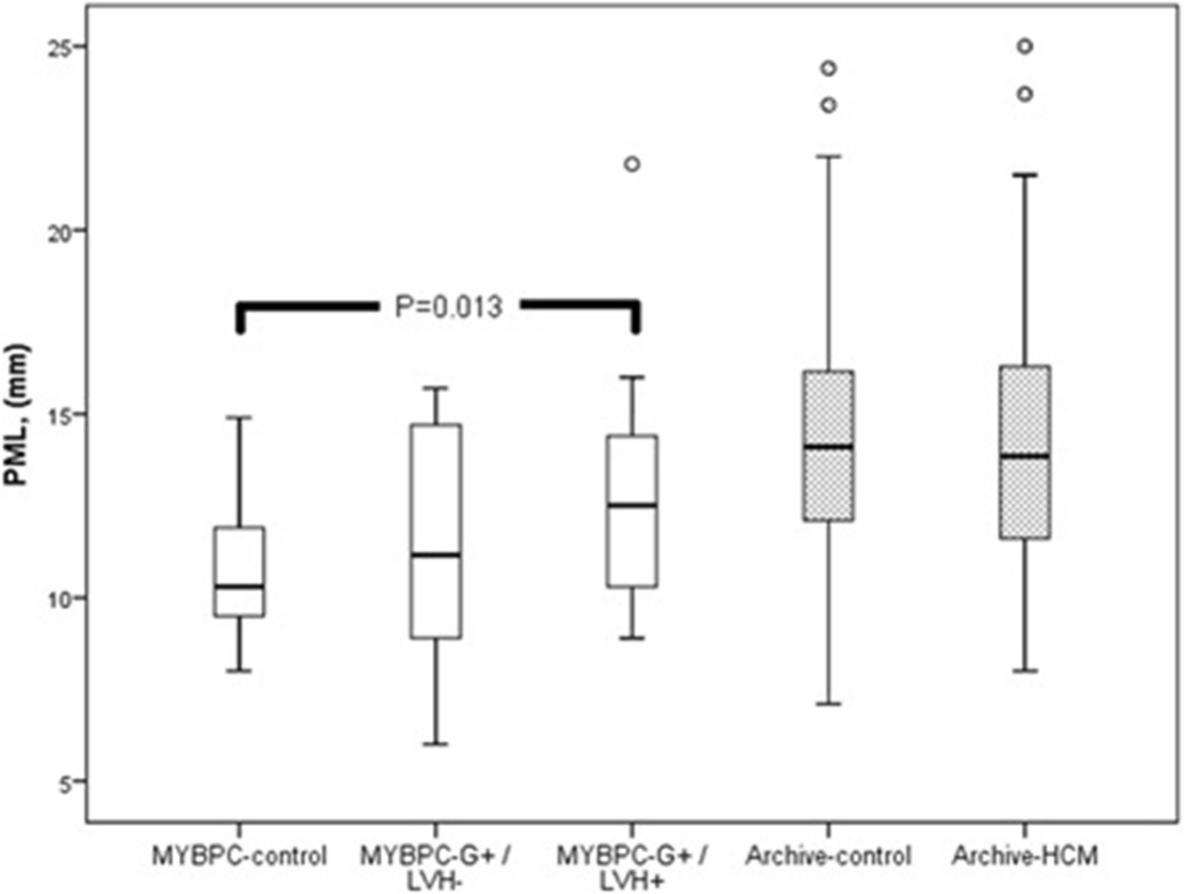
Posterior mitral valve leaflet (PML) lengths in study groups. The lower edge of the box presents 25th percentile and the upper edge 75th percentile. A line across the box is the median. The small circles present outliers. There is a significant difference between MYBPC genotype-positive/phenotype-positive (G+/LVH+) subjects and MYBPC-controls (*P* = 0.013)

Table 1 and Figs. 2 and 3 show the lengths of AML and PML in the study groups. In the MYBPC study, views were suitable for mitral valve leaflet measurements in 95 % of the study subjects for AML and 91 % for PML.

**Fig. 4:**
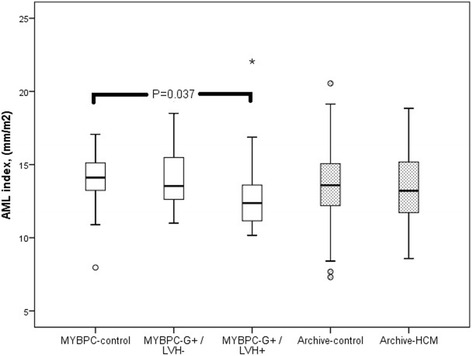
AML lengths indexed for body surface area (BSA) in study groups. The lower edge of the box presents 25th percentile and the upper edge 75th percentile. A line across the box is the median. The small circles present outliers and asterisk extreme value. There is a significant difference between MYBPC genotype-positive/phenotype-positive (G+/LVH+) subjects and MYBPC-controls (*P* = 0.037)

There was no difference in AML lengths between the MYBPC study subgroups, irrespective of indexing for BSA (Figs. 2 and 4). The indexed AML was even shorter in the MYBPC G+/LVH+ group than in other two groups. We also indexed AML for LVEDV and the difference between the MYBPC groups was insignificant (*P* = 0.460). There was one subject in both G+ subgroups in whom the length of AML exceeded + 2SD of the mean length of AML of the control group (Fig. 2).

**Fig. 5:**
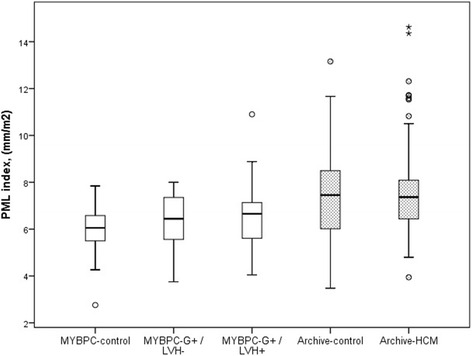
PML lengths indexed for body surface area (BSA) in study groups. The lower edge of the box presents 25th percentile and the upper edge 75th percentile. A line across the box is the median. The small circles present outliers and asterisk extreme value. There is no significant difference between the three and two groups, respectively

In contrast, there was a difference of borderline statistical significance in the length of PML between the MYBPC subgroups (Table 1, Fig. 3). PML was found to be significantly longer in the MYBPC G+/LVH+ group compared to the control group (12.8 ± 2.8 vs 10.6 ± 1.9 mm, *P* = 0.013) [Table 1]. When indexed for BSA, however, there was no significant difference in the PML length between the MYBPC G+/LVH+ group and the control group (6.6 ± 1.5 vs 5.8 ± 1.2 mm/m2, *P* = 0.066) (Table 1, Fig. 5). When PML was adjusted for age, gender and BSA, there was significant difference (*P* = 0.045) between the two groups. However, when further adjusted for LVM and LVEF, the difference in PML length was no longer significant between the MYBPC G+/LVH+ group and the control group (*P* = 0.195). We also indexed PML for LVEDV and the difference was insignificant between the MYBPC groups (*P* = 0.142).

There was no significant difference between the MYBPC G+/LVH- group and control group (11.1 ± 3.4 vs 10.6 ± 1.9 mm, *P* = 0.598) in PML lengths or BSA indexed PML lengths. As there was a difference in age and sex distribution between these two groups, we also selected age (±3 years) and sex matched control to each G+/LVH- subject and compared them pairwise. There was not any difference in mitral valve leaflet lengths, or in indexed leaflet lengths, in phenotype negative MYBPC3-Q1061X mutation carriers and controls (25.4 vs 26.0 mm, *P* = 0.726 for AML and 11.2 vs 10.6 mm, *P* = 0.680 for PML, and 14.3 vs 14.5 mm, *P* = 0.881 for BSA-indexed AML and 6.1 vs 5.9 mm, *P* = 0.729 for BSA-indexed PML, respectively) in matched pairwise comparison.

We found six subjects (19 %) in the MYBPC G+/LVH+ group and three subjects (20 %) in the MYBPC G+/LVH- group in whom the length of PML exceeded + 2SD of the mean length of PML of the control group.

In the Archive-group, AML could be measured in 95 % and PML in 88 % of the subjects. There was no difference in either AML lengths or PML lengths, or BSA indexed mitral valve leaflet lengths, between Archive-HCM patients and their matched control subjects (Table 1, Figs. 2 and 3). We tested AML and PML lengths after indexing for LVEDV and no significant difference between the Archive study groups was found (*P* = 0.328 for AML and *P* = 0.556 for PML). We also studied the mitral valve leaflet lengths including only the Archive-HCM patients with LVMWT 15 mm or more (instead of 13 mm or more) in the analysis, and there were no significant difference in mitral valve leaflet lengths compared to the controls (AML 25.8 ± 3.8 vs 25.1 ± 3.7 mm, *P* = 0.480, and PML 14.2 ± 3.4 vs 14.4 ± 3.6 mm, *P* = 0.585). There were 2 subjects in the Archive-HCM group in whom the length of AML exceeded + 2SD of the mean length of AML of the Archive-control group, and 3 subjects in whom the length of PML exceeded + 2SD of the mean length of PML of the Archive-control group.

### Relation of mitral valve length to left ventricular and clinical parameters

Table 2 shows associations between mitral valve leaflet lengths and clinical, echocardiographic and CMR findings in patients with MYBPC-Q1061X mutation (G+/LVH+ and G+/LVH- groups combined). In patients with MYBPC-Q1061X mutation (G+/LVH+ and G+/LVH- groups combined), PML length correlated with BSA (*r =* 0.405, *P* = 0.014), LVM (*r =* 0.379, *P* = 0.023), and reduced LVEF (*r =* −0.407, *P* = 0.014). AML length was associated with increased LVM only (*r =* 0.314, *P* = 0.036). There was no association between mitral valve leaflet length and age, gender or LVOT gradient.

**Table 2:** Spearman correlation coefficients between mitral leaflet length and clinical, echocardiographic and CMR findings in MYBPC G + and Archive-HCM groups

	MYBPC G+		Archive-HCM	
	AML	PML	AML	PML
age	.015	−.035	.050	.051
sex	.079	.309	−.079	.173
BSA	.098	.405*	−.035	.080
LVOT gradient	.257	−.241	N.A.	N.A.
LVMWT	.161	.152	−.020	.026
LVM	.314*	.379*	N.A.	N.A.
LVEDV	.196	.326	.301*	.060
LVESV	.215	.326	.348*	.109
LVEF	−.028	−.407*	−.250*	−.078

*AML* anterior mitral valve leaflet, *PML* posterior mitral valve leaflet, *BSA* body surface area, *LVOT gradient* left ventricular outflow tract gradient, *LVMWT* left ventricular maximal wall thickness, *LVM* left ventricular mass, *LVEDV* left ventricular end-diastolic volume, *LVESV* left ventricular end-systolic volume, *LVEF* left ventricular ejection fraction

*Significance *P* < 0.05

In the Archive-HCM group, AML length correlated with left ventricular volumes (LVEDV and LVESV) and negatively with LVEF. PML did not correlate with any clinical, echocardiographic or CMR parameters in the Archive-HCM group.

### Reproducibility of mitral valve measurements

In intraobserver analysis measurements, no difference between first and second measurements of the AML (25.4 ± 2.4 vs 25.3 ± 2.8 mm, *P* = 0.586) and PML (13.0 ± 3.4 vs 13.1 ± 4.4 mm, *P* = 0.784) lengths was found. Also the correlation between the measurements was almost perfect for AML (*r =* 0.949, *P* < 0.001) and for PML (*r =* 0.928, *P* < 0.001). In interobserver measurements, there were a systematic differences in AML (25.5 ± 4.6 vs 27.3 ± 6.5, *P* = 0.08) and PML (12.7 ± 3.1 vs 13.9 ± 3.0, *P* = 0.03) lengths between observers. In correlation analysis there were relatively good agreements between the measurements by two observers (*r =* 0.741, *P* < 0.001) for AML and (*r =* 0.657, *P* = 0.001) for PML.

## Discussion

### Principal findings

In the present study, patients with the *MYBPC3*-Q1061X mutation and LVH had slightly elongated posterior mitral valve leaflets (PMLs) compared to controls, but there was no difference between the groups when mitral valve leaflets were indexed for BSA, or adjusted for BSA, age, gender, LV mass and ejection fraction. In the MYBPC3 mutation carriers without LV hypertrophy, there was no difference in PML length compared to the MYBPC control group. In subjects with MYBPC-Q1061X mutation, including subjects with and without LVH, PML length correlated with BSA, LVM and reduced LVEF. No difference in AML length between the MYBPC3 mutation carriers, with or without hypertrophy and controls was found. In the non-genotyped unselected consecutive subjects with HCM of the Archive group, there was no difference in AML or PML lengths compared to control subjects.

### In the context of current literature

Recent studies indicate that CMR may be useful for establishing mitral valve morphology and pathology [20]. At the moment, however, there are no reference values for mitral valve leaflet lengths measured by CMR. So far, there are only four published CMR studies on mitral valve leaflet lengths in patients with HCM, or with HCM-causing sarcomeric mutations but no LVH [13–15]. These previous studies have shown elongated anterior and posterior mitral valve leaflets in 172 patients with non-genotyped HCM compared to control subjects [13], and elongated anterior mitral valve leaflets in subjects with HCM-causing mutations but no LVH [13–15]. In the study by Maron et al., 15 G+/LVH- subjects with mutations in MYBPC3, β myosin heavy chain and troponin T genes were studied [13]. In the studies by Captur et al., there were 73 and 40 G+/LVH- subjects with mutations in MYBPC3, MYH7, MYL2, MYL3, TNNT2, TNNI3, TPM1 and ACTC1 genes, respectively [14, 15]. In the recent study by Reant et al., 36 G+/LVH- subjects had mutations in the same genes [16] as in the studies by Captur.

In the present MYBPC study, in which all G+ subjects had a single *MYBPC*-Q1016X mutation, we found that some HCM patients with hypertrophic phenotype have elongated PML consistent with the concept that mitral valve leaflets may be elongated in HCM. However, in 80 non-genotyped unselected consecutive subjects with HCM of the Archive group, there was no difference in AML or PML lengths compared to control subjects, suggesting that mitral valve elongation is not a consistent feature of HCM in all its subpopulations. Furthermore, our study does not support the suggestion that mitral valve lengthening represents a primary phenotypic expression of the disease, as first, there was not any kind of difference in AML or PML lengths between the apparently healthy MYBPC3-Q1061X mutation carriers and controls; and second, the difference in PML length between the G+/LVH+ group and the control group was not significant after indexing for BSA, or when adjusted for BSA, age, gender, LV mass and ejection fraction.

### Possible mechanisms of elongated mitral valve leaflets

We found a positive association between PML length and BSA in MYBPC mutation carriers. Hence, mitral valve length, as many other structures of the heart, may be related to body size. However, PML length did not correlate with BSA in the Archive-HCM group. Furthermore, AML length did not correlate with BSA in the MYBPC G+ or Archive-HCM study groups. Consequently, studies in larger normal and patient populations are needed to confirm if mitral valve length is related to body size, and to define normal values of CMR derived mitral valve leaflet length.

In carriers of the MYBPC3-Q1061X mutation, PML length correlated with reduced EF and LVM, and AML length correlated with LVM. There was also a significant correlation between increased AML length and LV volume and reduced EF in subjects with HCM of the Archive group. Our findings suggest that mitral valve leaflet elongation may be related to LV remodeling in HCM. Cardiac remodeling, characterized by increased LVM, increased LV volumes and lower EF, may mechanically stretch the valve apparatus, leading to elongated mitral valve leaflets. In the Archive group of the present study, subjects with HCM had comparatively mild phenotype with moderate LVH and normal LV volumes, which may explain that, in contrast to the previous study [13], no significant elongation of mitral valve leaflets in these patients was observed.

LVOT obstruction is often associated with elongated mitral valve leaflets [12]. In the present study, significant LVOT obstruction was found only in one patient of MYBPC study, and consequently, the present study is not suitable for investigating the impact of LVOT obstruction on CMR derived mitral valve leaflet lengths. Respectively, low prevalence of LVOT obstruction (a history of myectomy in 2 % and LVOT flow velocity >2.75 m/s in 7.5 % of subjects) was found in our previous study on 306 Finnish patients with HCM, of which 35 had MYBPC3- Q1061X mutation [6].

It has been suggested that mitral valve abnormalities might be a primary phenotypic expression of HCM [13–15]. Sarcomeric mutations account for about 60 % of HCM cases, and variable sarcomeric genes, including troponin T, I and C, beta-and alpha myosin heavy chain, and myosin light-chain 2 genes, are expressed in interstitial cells of human heart valves [21]. According to current knowledge, however, MYBPC3 gene is expressed exclusively in cardiac muscle, and we have not found evidence that it is expressed in heart valves [22]. Consequently, primary mitral valve elongation might be evident only in HCM caused by mutations in genes that are expressed in heart valves. Furthermore, HCM-causing mutations in genes encoding sarcomere proteins induce LVH by at least two different mechanisms, incorporation of the mutant protein in the sarcomere, or haploinsufficiency due to absence of the mutant protein in the sarcomere [2, 23], respectively. Consequently, it is possible that not only the HCM-causing gene but also the mutation type may influence the disease expression in mitral valve. This highlights the importance to study mitral valve leaflet lengths in a sufficient number of subjects with identical HCM-causing mutations. Further larger studies are needed to clarify if mitral valve leaflets are elongated in subjects with mutations in variable HCM-associated genes and different type of gene variants.

### Study limitations and strengths

Although our MYBPC3-Q1016X genotype-positive study population is relatively small, it includes a moderate number of subjects with a single HCM-causing mutation. Thus, the confounding effect of variable disease-causing mutations on the valve length is avoided. MYBPC mutations are the most common cause of HCM, and represent 42 % percent of all HCM cases globally [5]. The Finnish founder mutation MYBPC-Q1016X, which is the most common genetic cause for HCM in Finland, is rare outside Finland. It might, however, be regarded to be representative of most MYBPC mutations, as like two thirds of all MYBPC3 mutations and consequently, about a third of all HCM-causing mutations globally, it leads to the production of a truncated protein and, consequently, to haploinsufficiency, characterized by absence of mutant protein in myocytes [22–24].

The number of mutation carriers without LVH was small in the present study, and they were younger and had a smaller BSA than the control subjects. However, not only the non-indexed but also the BSA-indexed mitral valve leaflet lengths in mutation carriers without LVH were similar to those of the control group, without even a trend for elongated leaflets in mutation carriers. Furthermore, when compared pairwise with age and sex matched controls, there was not any difference in mitral valve leaflet lengths in phenotype negative MYBPC3-Q1061X mutation carriers. On the basis of the present study, CMR-derived mitral valve length is not useful in identifying subjects with HCM-causing gene mutations but without LVH.

The number of consecutive unselected Archive HCM subgroup subjects of the present study was large and representative of all patients with HCM in the Kuopio University Hospital area. The subjects were well-matched with control subjects for age and gender, and in contrast to previous studies, mitral valve length was indexed for BSA, making it unlikely that the results of the Archive study are biased.

Finally, the reproducibility of mitral valve measurements was very high, with excellent intraobserver and acceptable interobserver variability, supporting the validity of the findings of the present study.

## Conclusions

In subjects with HCM caused by the Q1061X mutation in the *MYBPC3* gene, posterior mitral valve leaflets may be elongated, but mitral valve elongation does not constitute primary phenotypic expression of the disease. Instead, elongation of mitral valve leaflets in HCM seems to be associated with body size and left ventricular remodeling.

## Abbreviations

AML, anterior mitral valve leaflet; AML index, AML indexed for BSA; BSA, body surface area; CMR, cardiovascular magnetic resonance; HCM, hypertrophic cardiomyopathy; LVEDVI, left ventricular end-diastolic volume index; LVEF, left ventricular ejection fraction; LVESVI, left ventricular end-systolic volume index; LVM, left ventricular mass; LVMI, left ventricular mass index; LVMWT, left ventricular maximal wall thickness; LVOT gradient, left ventricular outflow tract gradient; MYBPC, myosin-binding protein C; NYHA, New York Heart Association functional class; PML, posterior mitral valve leaflet; PML index, PML indexed for BSA.
